# Human Resources for Cancer Control in Uttar Pradesh, India: A Case Study for Low and Middle Income Countries

**DOI:** 10.3389/fonc.2014.00237

**Published:** 2014-09-04

**Authors:** Maithili Daphtary, Sushma Agrawal, Bhadrasain Vikram

**Affiliations:** ^1^Radiation Research Program, National Cancer Institute, Rockville, MD, USA; ^2^Sanjay Gandhi Postgraduate Institute of Medical Sciences, Lucknow, India

**Keywords:** human resources, cancer control, low and middle income countries, Uttar Pradesh, India, cancer control planning

## Abstract

For addressing the growing burden of cancer in low and middle income countries, an important first step is to estimate the human resources required for cancer control in a country, province, or city. However, few guidelines are available to decision makers in that regard. Here, we propose a methodology for estimating the human and other resources needed in the state of Uttar Pradesh (UP), India as a case study. Information about the population of UP and its cities was obtained from http://citypopulation.de/. The number of new cancer cases annually for the commonest cancers was estimated from GLOBOCAN 2008^1^. For estimating the human resources needed, the following assumptions were made: newly diagnosed cancer patients need pathology for diagnosis and for treatment surgery, chemotherapy, and/or radiotherapy. The percentage of patients requiring each of those modalities, their average lengths of stay as in-patients, and number of in-patient oncology beds were estimated. The resources already available in UP were determined by a telephone survey and by searching the websites of radiation therapy centers and medical colleges. Twenty-four radiation oncologists at 24 cancer centers in 10 cities responded to the survey. As detailed in this manuscript, an enormous shortage of human resources for cancer control exists in UP. Human resources are the key to diagnosing cancers early and treating them appropriately. Addressing the shortage will not be easy but we hope that the methodology described here can guide decision makers and form a framework for discussion among the various stakeholders. This methodology is readily adaptable to local practices and data.

## Introduction

Low and middle income countries (LMIC) face an increasing burden of cancer (1, 2). To effectively address this problem, cancer control planning at the country, state, city, and community level is needed. However, the scarcity of cancer registries and lack of guidelines for cancer control planning/capacity building make this a difficult undertaking for stakeholders.

Several recent publications including the WHO report (2014) and the Global Burden of Disease reports (Lancet 2012) have discussed in detail the magnitude and the reasons for the growing burden of cancer in LMIC in general and India in particular^2^. The number of new cancer cases in India was 0.95 million in 2008 and projected to increase to 1.7 million in 2035. The incidence of cancer in India is lower than in Western nations, but the mortality is higher suggesting low health service effectiveness. Probably for the same reason, while the incidence of cancer among persons living in rural areas is half that of urban dwellers, their age-adjusted standardized mortality rates from cancer are similar. Contributory factors to the growing burden of cancer include longer life spans, growing urbanization and industrialization, use of tobacco, sedentary lifestyles, etc. Strategies for preventing cancers are, therefore, a high priority for India. At the same time, for decreasing the deaths and suffering from cancers, the health systems for cancer control must be strengthened to facilitate the early diagnosis and effective treatment of those cancers that cannot be prevented for the foreseeable future. Human resources are obviously a key component of such a strategy.

Here, we propose a method for estimating the human and other resources necessary for treating the most common cancers in LMIC and compare them to those available, using the state of Uttar Pradesh (UP) in India as a case study. UP with a population of approximately 200 million is the most populous state in India and probably the most populous country subdivision on the planet. It is diverse in terms of urbanization, distribution of wealth, resources, and access to education and healthcare and has a large, well-developed transportation network. Importantly, oncologists and advocates in UP are interested in bringing this growing life-threatening cancer crisis to the attention of politicians and policy makers who can mobilize the funding for building the infrastructure – both human and physical – needed for cancer prevention and control in UP.

## Materials and Methods

The population of India, UP, and its various cities was obtained from http://citypopulation.de/. The number of new cancer cases annually and the major types of cancers in India was obtained from GLOBOCAN 2008^1^. GLOBOCAN does not report data for states or cities. In the absence of UP cancer registry data, we assumed that the proportion of the various kinds of cancers in UP was the same as all of India. Thus, based on the population of UP and the number of new cancer cases in India, the number of new cancer cases for UP was estimated (Table 1). Estimates can be revised if and when more accurate data become available.

**Table 1:** **The most common cancers in Uttar Pradesh for men and women based upon GLOBOCAN 2008 data**.

	Both sexes	Rank	Men	Rank	Women	Rank
All cancers excluding non-melanoma skin cancer	160296		72659		87637	
Gynecological	28934	1			28934	1
Head and neck	26080	2	18359	1	7721	3
Breast	19470	3			19470	2
Hematological malignancies	12026	4	7301	3	4725	4
Lung	9894	5	7942	2	1952	8
Esophagus	8126	6	4867	5	3259	5
Urological	7130	7	6040	4	1090	11
Colorectal	6162	8	3406	7	2756	6
Stomach	5923	9	3561	6	2362	7
Brain, nervous system	3689	10	2211	9	1478	10
Liver	3403	11	2452	8	951	12
Gallbladder	2916	12	1001	10	1915	9
Pancreas	1513	13	858	11	655	13
Melanoma of skin	160	14	85	12	75	14

### Estimating the human and other resources needed for treating new cancer cases in UP

For estimating the human and other resources needed for treating the various kinds of cancers, several specialty societies and organizations were consulted. Except for the International Atomic Energy Agency (IAEA) of the United Nations^3^, most could offer no official guidelines. Therefore, numerous colleagues who are active in those fields were consulted informally and are listed in the Section “Acknowledgments.” Based upon their feedback and opinions, the following assumptions were made and used for our calculations, which can be readily revised if and when more accurate data become available (or to confirm better to local practices):

- Newly diagnosed cancer patients need pathology review of their tissue for diagnosis. They also require surgery, chemotherapy, and/or radiation therapy for treatment. The percentage of patients requiring each of those therapeutic modalities and the average length of stay as in-patients were estimated for the most common cancers in UP and are shown in Table 2.
- The number of specialists needed was estimated based upon the number of patients requiring surgery, chemotherapy, and/or radiation therapy, as well as pathology annually. For LMIC, rather than implementing separate medical and radiation oncology training tracks, the IAEA recommends training radiation/clinical oncologists who can prescribe both radiation and chemotherapy for common solid cancers. The number of radiation/clinical oncologists needed is estimated at 5 per 1000 cancer patients. The number of pathologists needed is estimated at 2 per 1000 cancer patients, recognizing that most of them do not concentrate solely on cancer. The number of surgical oncologists needed is based on the number of cancer patients requiring surgery, assuming that each surgical oncologist performs two surgeries per day, 5 days per week for 48 weeks per year. The number of gynecological oncologists, urological oncologists, neurological oncologists, and hematologist-oncologists needed is 2 per 1000 patients with gynecological, urological, neurological, and hematological malignancies, respectively. Two palliative care specialists will be needed for each 1000 new cancer patients.
- Many cancer patients require hospitalization for diagnosis and/or treatment of cancer and its complications. The number of oncology beds needed per day is the sum of the number of beds needed for surgery, chemotherapy, and radiation therapy for newly diagnosed cancer patients with the most common cancers. An oncology ward is a 24-bed in-patient unit for only oncology patients that should be staffed by 15 oncology nurses, 4 oncopharmacists, and 6 pharmacy technicians.
- Many cancer patients require radiotherapy; therefore, appropriately equipped facilities are needed along with well-trained radiation oncology staff. The radiation oncology staff needed includes radiation/clinical oncologists (as discussed earlier) and for every 1000 patients requiring radiation therapy, 12 radiation therapy technicians, 4 medical physicists, 1 linear accelerator (linac) engineer, and 4 radiation therapy nurses. The minimum radiation therapy equipment requirements for every 1000 patients requiring radiation therapy are at least 1 for each of the following: megavoltage teletherapy unit (linac or cobalt), brachytherapy unit, CT Simulator, treatment planning computer system, and dosimetry/Quality Assurance package. If there is only 1 MV teletherapy unit per 1000 radiation therapy patients, it should be operated nearly non-stop, albeit with regularly scheduled downtime for preventive maintenance and quality assurance, otherwise a minimum of two such units are needed.
- Each city, in order to ensure coverage if one person leaves or goes on vacation should have at least two professionals of each kind.

**Table 2:** **The percentage of requiring patients various kinds of treatment and their average length of stay (ALOS) in hospital (in days)**.

	Percent of patients requiring surgery (ALOS)	Percent of patients requiring chemotherapy (ALOS)	Percent of patients requiring radiotherapy (ALOS)
Gynecological cancers	57 (6.5)	67 (3)	40 (5)
Cervix uteri	20 (5)	80 (3)	80 (5)
Corpus uteri	80 (5)	20 (3)	20 (5)
Ovary	70 (9)	100 (3)	20 (5)
Head and neck cancers	44 (7)	66 (3.5)	71 (5)
Larynx	50 (9)	50 (3)	75 (5)
Lip and oral cavity	40 (9)	80 (3)	80 (5)
Nasopharynx	0 (0)	100 (3)	100 (5)
Other pharynx	40 (9)	80 (3)	80 (5)
Thyroid	90 (7)	20 (5)	20 (5)
Hematological malignancies	0 (0)	100 (6.5)	33 (5)
Hodgkin’s lymphoma	0 (0)	100 (5)	40 (5)
Leukemia	0 (0)	100 (7)	20 (5)
Multiple myeloma	0 (0)	100 (7)	20 (5)
Non-Hodgkin’s lymphoma	0 (0)	100 (7)	50 (5)
Urological cancers	74 (8)	63 (3.5)	41 (5)
Bladder	100 (9)	50 (3)	50 (5)
Kidney	75 (9)	50 (3)	20 (5)
Prostate	20 (9)	50 (3)	65 (5)
Testis	100 (5)	100 (5)	30 (5)
Brain and nervous system cancers	100 (9)	100 (3)	100 (5)
Breast cancers	100 (5)	100 (3)	100 (5)
Colorectal cancers	70 (9)	90 (3)	25 (5)
Gallbladder cancers	33 (9)	66 (3)	50 (5)
Kaposi’s sarcoma	0 (0)	70 (3)	50 (5)
Liver cancers	5 (10)	20 (3)	20 (5)
Lung cancers	25 (10)	50 (3)	90 (5)
Melanoma of the skin	100 (3)	50 (3)	50 (5)
Esophagus cancers	20 (9)	90 (3)	90 (5)
Pancreas cancers	10 (10)	50 (5)	50 (5)
Stomach cancers	33 (5)	66 (3)	50 (5)

*It was assumed that for surgery or chemotherapy, all patients required hospitalization initially whereas for radiotherapy only one-quarter required hospitalization*.

### Estimating the human and other resources already available in UP

The radiation therapy resources available (radiation/clinical oncologists, radiation therapy staff, and radiation therapy equipment) were determined by telephone survey and searching websites of radiation therapy centers and medical colleges in the state of UP. A list of cancer centers with radiation therapy facilities in UP was obtained from the database of oncology centers in the Department of Radiotherapy, Sanjay Gandhi Postgraduate Institute of Medical Sciences, Lucknow. Twenty-four radiation oncologists at 13 government and 11 private cancer centers in 10 cities were contacted for a telephone survey and all 24 responded to the survey. The telephone survey was conducted by one of the authors (SA) who was also 1 of the 24 respondents.

The number of specialists in allied departments (urology, neurosurgery, gynecology, etc.) was also estimated by telephone survey and from websites of the government and private cancer centers. The number of beds available for cancer patients was similarly estimated from those websites. No attempt was made to document the number of oncopharmacists and pharmacy technicians as those specializations did not exist in UP.

## Results

In the year 2008, there were 948,858 new cases of cancer in India and as shown in Table 1, there were 160,296 new cases in UP. Extrapolating those numbers to the 10 cities in UP with radiotherapy centers (population range 0.5–2.8 million) yielded the estimated number of new cancer patients in each city and the numbers requiring surgery, chemotherapy, and/or radiotherapy, as well as the required number of oncology beds in each city. It is evident that a vast proportion of the population of UP lives outside those 10 cities, therefore, the numbers needed for UP as a whole far exceed the numbers needed for the 10 cities.

Table 3 compares the number of specialists needed in UP with those available. A comparison of the number of specialists needed and available reveals that for UP state, there is a shortage of 715 clinical/radiation oncologists, 142 pathologists, 115 surgical oncologists, 34 gynecological oncologists, and 18 hematologist-oncologists. “Gastro-surgeons” are a recognized specialty in UP; the bulk of their practice consists of gastrointestinal cancer surgery; therefore, the available 21 gastro-surgeons were added to the surgical oncologists for a total of 42 available surgical oncologists.

**Table 3:** **Number of oncologists needed versus those available for UP and its 10 cities with radiation therapy centers based upon GLOBOCAN 2008 data**.

	Hematologist-oncologists		Number requiring surgery	Surgical oncologists		Radiation/clinical oncologists		Urologic oncologists		Gynecologic oncologists		Neurologic oncologists		Pathologists	
	Needed	Available		Needed	Available	Needed	Available	Needed	Available	Needed	Available	Needed	Available	Needed	Available
UP	25	7	74860	157	21	802	87	20	20	58	24	20	26	321	179
Agra	2^a^	0	591	2	0	7	4	2^a^	0	2^a^	2	2^a^	2	3	11
Aligarh	2^a^	0	327	2^a^	0	4	3	2^a^	0	2^a^	2	2^a^	0	2	16
Allahabad	2^a^	1	419	2^a^	1	5	9	2^a^	1	2^a^	2	2^a^	2	2	25
Bareilly	2^a^	0	337	2^a^	1	4	6	2^a^	0	2^a^	2	2^a^	1	2	15
Benares	2^a^	1	451	2^a^	5	5	9	2^a^	4	2^a^	2	2^a^	4	2	15
Gorakhpur	2^a^	0	252	2^a^	1	3	5	2^a^	0	2^a^	3	2^a^	2	2	16
Jhansi	2^a^	0	191	2^a^	0	3	2	2^a^	0	2^a^	2	2^a^	0	2^a^	16
Kanpur	2^a^	0	1038	3	1	12	10	2^a^	1	2^a^	2	2^a^	3	5	15
Lucknow	2	3	1056	3	6	12	28	2^a^	10	2^a^	4	2^a^	10	5	37
Noida	2^a^	2	241	2^a^	6	3	11	2^a^	4	2^a^	3	2^a^	2	2	13

*^a^At least two are required in each city*.

Table 4 shows the number of oncology beds and professionals (nurses, oncopharmacists, and pharmacy technicians) needed and available. Comparing the numbers needed to those available reveals that, in UP state, there is a shortage of 2018 oncology beds, 1582 oncology nurses, 313 palliative care specialists, 484 oncopharmacists, and 726 pharmacy technicians.

**Table 4:** **Number of oncology beds, nurses, pharmacy staff, and palliative care specialists needed versus those available for UP and its 10 cities with radiation therapy centers based upon GLOBOCAN 2008 data**.

	Number of oncology beds		Number requiring chemotherapy	Oncopharmacists		Pharmacy technicians		Palliative care specialists		Oncology nurses	
	Needed	Available		Needed	Available^b^	Needed	Available^b^	Needed	Available	Needed	Available
UP	2892	875	117172	484	0	726	0	321	8	1815	233
Agra	22	50	925	4	0	6	0	3	0	15	10
Aligarh	13	35	512	4	0	6	0	2	0	15	7
Allahabad	16	90	656	4	0	6	0	2	0	15	13
Bareilly	13	50	528	4	0	6	0	2	0	15	10
Benares	18	100	706	4	0	6	0	2	2	15	20
Gorakhpur	10	85	394	4	0	6	0	2	0	15	15
Jhansi	8	40	298	4	0	6	0	2^a^	0	15	8
Kanpur	40	150	1625	8	0	12	0	5	0	30	50
Lucknow	41	175	1653	8	0	12	0	5	5	30	75
Noida	9	100	377	4	0	6	0	2	1	15	25

*^a^At least two are required in each*.

*^b^There is no oncology specialization for pharmacy technicians and pharmacists*.

Tables 5 and 6 show the radiation oncology staff and equipment that is needed and available.

**Table 5:** **Radiation therapy (RT) staff needed versus available for UP and its 10 cities with radiation therapy centers based upon GLOBOCAN 2008 data**.

	Number requiring radiotherapy	Radiation/clinical oncologists		RT technicians		Medical physicists		Linac engineers		RT nurses	
		Needed	Available	Needed	Available	Needed	Available	Needed	Available^b^	Needed	Available^b^
UP	94808	802	87	1138	83	380	38	95	0	380	0
Agra	748	7	4	9	5	3	2	2^a^	0	3	0
Aligarh	415	4	3	5	2	2	1	2^a^	0	2	0
Allahabad	531	5	9	7	11	3	5	2^a^	0	3	0
Bareilly	427	4	6	6	5	2	3	2^a^	0	2	0
Benares	571	5	9	7	12	3	4	2^a^	0	3	0
Gorakhpur	319	3	5	4	6	2	2	2^a^	0	2	0
Jhansi	241	3	2	3	2	2^a^	1	2^a^	0	2^a^	0
Kanpur	1315	12	10	16	8	6	3	2	0	6	0
Lucknow	1338	12	28	17	20	6	11	2	0	6	0
Noida	305	3	11	4	12	2	6	2^a^	0	2	0

*^a^At least two are required in each*.

*^b^There is no specialization for RT nurses or Linac engineers in UP*.

**Table 6:** **Radiation therapy equipment needed versus available for UP and its 10 cities with radiation therapy centers based upon GLOBOCAN 2008 data**.

	Megavoltage teletherapy units		Brachytherapy units		CT simulators		Treatment planning computer systems		Dosimetry/QA packages	
	Needed	Available	Needed	Available	Needed	Available	Needed	Available	Needed	Available
UP	190	26	95	17	95	11	95	19	95	0
Agra	2	1	1	1	1	0	1	1	1	0
Aligarh	1	1	1	1	1	0	1	1	1	0
Allahabad	2	3	1	2	1	2	1	2	1	0
Bareilly	1	2	1	1	1	1	1	1	1	0
Benares	2	4	1	2	1	1	1	2	1	0
Gorakhpur	1	2	1	1	1	0	1	1	1	0
Jhansi	1	1	1	0	1	0	1	0	1	0
Kanpur	3	3	2	2	2	2	2	2	2	0
Lucknow	3	6	2	5	2	3	2	6	2	0
Noida	1	3	1	2	1	2	1	3	1	0

Comparing the number of radiation oncology staff needed to that available (Table 5) reveals that in UP state, there is a shortage of 715 clinical/radiation oncologists, 1055 radiotherapy technicians, 380 radiotherapy nurses, 342 medical physicists, and 95 linac engineers. Comparing the radiation oncology equipment needed to that available (Table 6) reveals that in UP, there is a shortage of 164 MV teletherapy units, 78 brachytherapy units, 84 CT simulators, 76 treatment planning computer systems, and 95 dosimetry/quality assurance packages.

## Discussion

We found that an enormous shortage of human and other resources for cancer control exists in the state of UP (Tables 3–6). In fact, the shortage may be even worse than the tables indicate, because we estimated the resources needed from year 2008 data whereas, according to GLOBOCAN, the number of new cancer cases in India is projected to increase from 948,858 in the year 2008 to 1,220,000 by 2016, an increase of almost 30%. Assuming that the same is true for UP, the resources needed in the year 2013 would be about 20% greater, and in the year 2016 about 30% greater, than shown in Tables 3–6 (our telephone survey for estimating the resources already available was conducted in 2013).

As a part of our survey, we learned that at present only 18 physicians enter radiation/clinical oncologist training programs in UP annually. More than 800 (probably an underestimate) are needed as shown in Table 3. Unless steps are taken to dramatically increase the training opportunities and incentives, it may take nearly a century to address the shortage.

A previous effort to address the shortages included a modest proposal in India’s 11th plan (2007–2012) of the National Cancer Control Program (accessed Dec 26, 2013)^4^ that there should be at least one radiation oncology center for every four districts. With its 75 districts, UP would accordingly require 19 centers by the year 2012, but only one was added between 2007 and 2012.

Our findings illustrate that the delivery of affordable and equitable cancer care remains one of India’s greatest public health challenges. Specific figures for UP are not available but public expenditure on cancer in India remains below US$10 per person (compared with more than US$100 per person in high-income countries), and overall public expenditure on health care is still only slightly above 1% of gross domestic product (3). The crucial issues of infrastructure, public insurance schemes, the need to develop new political mandates and authority to set priorities, the necessity to greatly improve the quality and delivery of cost-effective cancer care are inextricably linked with the shortage of human resources necessary for the prevention and control of cancer.

Addressing this shortage will not be easy, but we hope that the data provided in this paper can form a framework for discussion among the various stakeholders. It is noteworthy that the total population of the 10 major cities with radiotherapy-containing cancer centers accounted for less than one-tenth of the population of UP. Establishing additional cancer centers will therefore be necessary in those parts of UP that are not close to any of the 10 cities. At the same time, it will be necessary to strengthen the existing cancer centers because at least some of them already appear to service quite a large number of patients from outside the cities that the cancer centers are located in. In the UP state capital Lucknow, there were just over an estimated 2000 new cancer cases in 2008, projected to increase to about 3000 by the year 2013. However, our 2013 telephone survey revealed that during the preceding 12 months, more than 8000 new cancer patients were seen at the various hospitals in Lucknow. We did not have the resources to try and determine how many of those patients originated from outside Lucknow. This discrepancy, in fact, highlights the glaring lack of the urgent need for establishing cancer registries in UP for capturing more accurate and granular data on the incidence and outcomes of cancers.

### Cancer prevention and early detection

Cancer prevention is, of course, preferable to cancer control. Among the cancers most common in UP, many future cancers of the uterine cervix may be preventable by vaccines (4) while many cancers of the mouth, throat, and lung may be prevented by effective tobacco control (accessed December 16, 2013)^5^.

For some cancers, such as the uterine cervix, early detection and treatment are also feasible. Visual inspection with acetic acid followed by the immediate treatment of suspicious lesions has been proposed as a possibly effective strategy suitable for widespread implementation in LMIC (5). Analogous to the methodology described in this paper, we also estimated the human resources needed in different countries for implementing such a population-wide intervention. We focused on those countries where cervical cancer was among the top five cancers among women and the results are available at http://rrp.cancer.gov/programsResources/hrn_cervical_cancer_screening.htm. In the case of UP, once again extrapolating from India as a whole, we found that approximately 2000 health workers would be needed to screen on three occasions for pre-invasive cervical cancer, and treat, when indicated, all women between the ages of 30 and 45 years. That number would include 400 supervisors (usually physicians) and 1600 other health workers (specially trained non-physicians such as nurses and midwives).

## Global Implications

Interventions for detecting cancers early and treating them appropriately are crucial components of cancer control planning. Human resources are the key but, unfortunately, are often neglected in LMIC. In planning new radiotherapy facilities, the major focus may be on the buildings and equipment while only a token number of staff are trained and/or hired. This results in chronically understaffed and poorly maintained facilities, leading to poor patient outcomes and low staff morale. The cost of treating each patient escalates because after making the substantial investment in buildings and equipments, fewer patients are treated than could have been in an adequately staffed facility.

One of the reasons that human resources are neglected may simply be the lack of guidance available to decision makers. We hope that the methodology described in this paper can provide a framework for discussion among the stakeholders interested in cancer control in a country, state, city, or community. As we have tried to emphasize, the methodology is readily adaptable to local practices and data.

## Conflict of Interest Statement

Drs. Maithili Daphtary and Bhadrasain Vikram contributed to this manuscript in their personal capacity and its contents may not reflect the official positions of the National Institutes of Health or the US Department of Health and Human Services.

## Footnotes

^1^http://globocan.iarc.fr accessed on October 15, 2013

^2^http://www.thelancet.com/series/cancer-burden-and-health-systems-in-india

^3^http://www-pub.iaea.org/MTCD/publications/PDF/pub1296_web.pdf

^4^http://mohfw.nic.in/index1.php?lang=1&level=2&sublinkid=323&lid=323

^5^http://www.who.int/cancer/nccp/en/
